# Krebs von den Lungen 6 decreased in the serum and muscle of GNE myopathy patients

**DOI:** 10.1111/neup.12703

**Published:** 2020-11-22

**Authors:** Takashi Kurashige, Tetsuya Takahashi, Yoshito Nagano, Kazuma Sugie, Hirofumi Maruyama

**Affiliations:** ^1^ Department of Neurology National Hospital Organization Kure Medical Center and Chugoku Cancer Center Kure, Hiroshima Japan; ^2^ Department of Clinical Neuroscience and Therapeutics, Division of Applied Life Science Hiroshima University Institute of Biomedical and Health Sciences Hiroshima Japan; ^3^ Department of Neurology Nara Medical University School of Medicine Kashihara, Nara Japan

**Keywords:** GNE myopathy, hyposialylation, KL‐6, MUC1, rimmed vacuoles

## Abstract

UDP‐*N*‐acetylglucosamine 2‐epimerase/*N*‐acetylmannosamine kinase (GNE) is necessary for sialic acid biosynthesis. GNE myopathy is caused by a defect in *GNE*, and hyposialylation is a key factor in the pathomechanism of GNE myopathy. Although candidates for evaluating hyposialylation have been reported, it is difficult to measure them in routine clinical practice. Sialylation is necessary for synthesis of various glycoproteins, including Krebs von den Lungen‐6 (KL‐6)/mucin 1 (MUC1). Here we report that KL‐6/MUC1 is decreased in GNE myopathy. We observed that KL‐6 levels were decreased in the serum of patients with GNE myopathy, and that KL‐6 and MUC1‐C were also decreased in muscle biopsy specimens from these patients. An immunofluorescent study revealed that KL‐6 and MUC1‐C were not present in the sarcolemma but were, instead, localized in rimmed vacuoles in specimens from patients with GNE myopathy. KL‐6 is already used to detect lung diseases in clinical practice, and this glycoprotein may be a novel candidate for evaluating hyposialylation in GNE myopathy.

## INTRODUCTION

UDP‐*N*‐acetylglucosamine 2‐epimerase/*N*‐acetylmannosamine kinase (GNE) myopathy (GNEM) is characterized clinically by distal dominant muscle atrophy and weakness and pathologically by rimmed vacuoles (RVs), which are accumulations of autophagic vacuoles and various proteins.^1^, ^2^ The causative gene is *GNE*, which encodes the bifunctional enzyme UDP‐*N*‐acetylglucosamine 2‐epimerase/*N*‐acetylmannosamine kinase (GNE/MNK).^3^, ^4^ This cytosolic enzyme is essential for sialic acid biosynthesis,^5^, ^6^ and GNE/MNK enzymatic activities are reduced to 70–90% in cells transfected with mutant *GNE*.^7^ GNEM model mice can be effectively treated with oral sialic acid metabolites.^8^ Thus, hyposialylation is a key factor in the pathomechanism of GNEM.^2^ Sialic acid is a necessary component of various glycoproteins, and recent reports have demonstrated that the Thomsen–Friedenreich antigen and transferrin are candidates for evaluating hyposialylation in GNEM.^2^, ^9^, ^10^, ^11^ These molecules are measured by mass spectroscopy^9^ or transferrin isoelectric focusing,^10^ but these methods are too complicated for use in routine clinical practice.

The genes encoding some glycoproteins, such as mucins and alpha‐dystroglycan, have variable number tandem repeat (VNTR) domains. Sialosides are attached to protein domains of these glycoproteins encoded by VNTRs. The glycoproteins are then translocated to the cell surface, where they are involved in various cellular functions.^5^, ^12^, ^13^, ^14^ Defects in glycosyltransferases result in hypoglycosylation of *VNTR*‐encoded protein regions; this is the pathomechanism of alpha‐dystroglycanopathy.^12^, ^13^ Similarly, hypoglycosylation of *VNTR*‐encoded protein regions due to *GNE* mutations may also be associated with the pathomechanism of GNEM.

Mucin 1 (MUC1), a transmembrane‐type mucin, consists of a C‐terminal subunit that includes a transmembrane domain (MUC1‐C), as well as an N‐terminal subunit that includes the *VNTR*‐encoded polypeptide (MUC1‐N). The MUC1 precursor protein is autocleaved to produce MUC1‐C and MUC1‐N, and these subunits form the MUC1 complex, similar to dystroglycan.^13^, ^14^ The molecular weight of MUC1 is approximately 200 kDa, while that of MUC1‐C is approximately 50 kDa. The biological half‐life of MUC1 is around 24 h.^14^ The MUC1 complexes are glycosylated in the Golgi apparatus and translocated to the cell surface.^15^ MUC1 expression levels are higher in epithelial tissues than in skeletal muscles. MUC1 complexes are larger than other proteins, including dystrophin‐glycoprotein complexes. MUC1 complexes hydrate cell surfaces and inhibit cell–cell and cell–extracellular matrix interactions in normal and malignant contexts, thus protecting epithelial cells against microorganisms. The amount of MUC1 complexes increases according to the glycosylation levels, and hypoglycosylation of MUC1 leads to release of MUC1‐C from MUC1 complexes and the degradation of MUC1‐C.^16^

Krebs von den Lungen‐6 (KL‐6) is a sialo‐carbohydrate antigen detected by a mouse monoclonal antibody that recognizes the sialylated sugar chain of *VNTR*‐encoded regions of human MUC1‐N.^17^, ^18^, ^19^ KL‐6 is found only in humans and apes,^20^ and *N*‐acetylneuraminic acids (NeuAc) account for approximately 30% of the KL‐6 sugar content.^18^ The primary source of serum KL‐6 is type II pneumocytes, especially regenerating type II pneumocytes.^17^ KL‐6 causes fibrotic processes of interstitial pneumonia because KL‐6 itself is a chemotactic factor for fibroblasts and promotes proliferation and survival of fibroblasts.^19^, ^21^ KL‐6 has been developed as an important serum biomarker for various interstitial lung diseases.^17^, ^22^, ^23^ In skeletal muscle, normal KL‐6 expression is weaker than in epithelial cells, but GNE/MNK functions as a cytosolic enzyme for sialic acid biosynthesis in all cells. *GNE* deficits may cause hypoglycosylation of *VNTR*‐encoded regions of MUC1‐N and a subsequent decrease in MUC1 complexes and KL‐6 sialo‐carboxylate antigens.

Here we demonstrate the expression status of KL‐6 and MUC1 in serum and muscle biopsy specimens from patients with GNEM. Biosynthesis of KL‐6 and MUC1 may be associated with sialic acid metabolism in such patients.

## MATERIALS AND METHODS

### Cases

Fifty‐three patients with available clinical records and serum samples were included in this study. Patients presenting with pneumonia at their diagnosis were excluded because their serum KL‐6 levels were likely elevated due to their interstitial pneumonia.^17^, ^22^, ^24^ We examined eight patients with GNEM who were diagnosed clinically, pathologically, and genetically but were not on medication, including whey proteins. Patients with GNE myopathy, except for patients 7 and 8, had the p.V603L homozygous *GNE* mutation. Patient 7 had the homozygous p.D207L mutation and patient 8 had the p.G167R/p.D207L compound heterozygous *GNE* mutation. The control subjects were six patients with rimmed vacuolar distal myopathy (DM) without *GNE* mutations (other RVM), 13 patients with sporadic inclusion body myositis (sIBM), 20 patients with idiopathic inflammatory myopathy (IIM) including polymyositis and autoimmune‐mediated necrotizing myopathy, and nine individuals with morphologically normal control (NC) muscles. Patients with sIBM and IIM fulfilled typical clinical, electrophysiological, and histopathological criteria.^25^, ^26^, ^27^, ^28^ Their laboratory data, which were obtained during routine blood testing at the time of muscle biopsy or before treatment, were evaluated with a PicoLumi II automatic ECLIA system (Eidia, Japan) according to the manufacturer's instructions. The characteristics of the study subjects are summarized in Table 1 and Table S1. Ileum and skeletal muscle tissues for investigation of the organ profile of skeletal muscle for Western blotting were prepared from an autopsy case without neuromuscular, gastrointestinal, or lung diseases.

**Table 1:** Clinical characteristics of patients in this study

		GNEM (n = 8)	GNE‐negative DM (n = 6)	sIBM (n = 13)	IIM without IP (n = 20)	NC (n = 9)
Age (mean ± SD years)		34.8 ± 12.9	41.2 ± 11.0	66.0 ± 13.6	60.5 ± 12.8	30.1 ± 6.8
Gender (M:F)		6:2	2:4	9:4	8:12	5:4
Disease duration (mean ± SD years)		7.6 ± 2.8	19.8 ± 13.0	3.1 ± 1.3	1.3 ± 1.5	n.a.
Biopsy site						
Deltoid		0	1	0	0	0
Biceps brachii		3	5	5	7	4
Triceps brachii		0	0	0	2	0
Vastus lateralis		1	0	8	11	3
Tibialis anterior		4	0	0	0	0
Fiburalis brevis		0	0	0	0	2
Laboratory data (mean ± SD)	CK (IU/L)	635.1 ± 560.6	696.8 ± 816.1	802.2 ± 474.5	2765.0 ± 3496.2	3675.2 ± 7741.8
	LDH (IU/L)	276.5 ± 107.9	320.5 ± 165.3	421.7 ± 208.9	896.8 ± 829.5	519.1 ± 654.5
	KL‐6 (IU/L)	173.0 ± 46.8	265.8 ± 29.1	233.8 ± 51.1	287.4 ± 105.5	231.9 ± 54.3

CK, creatine kinase; DM, distal myopathy; F, female; GNEM, GNE myopathy; IIM, idiopathic inflammatory myopathies; IP, interstitial pneumonia; KL‐6, Krebs von den Lungen‐6.; LDH, lactate dehydrogenase; M, male; NC, morphologically normal control; sIBM, sporadic inclusion body myositis.

The study protocol was approved by the ethics committee of the Hiroshima University Institute of Biomedical and Health Sciences, and written informed consent was obtained from all patients.

### Muscle biopsy specimens

All muscle biopsy specimens and tissues from the autopsy case were frozen in liquid nitrogen‐cooled isopentane. The pathological diagnosis was confirmed with routine histochemistry and immunohistochemistry. Serial transverse 7‐μm‐thick sections were stained with hematoxylin and eosin, modified Gomori trichrome, and a battery of histochemical stains for pathological diagnosis.

### Immunohistochemistry

Serial transverse 7‐μm‐thick frozen sections from muscle biopsy specimens from each case were stained by modified Gomori staining and immunohistochemistry. Frozen sections for immunohistochemistry were incubated overnight at 4°C with a primary mouse monoclonal antibody against KL‐6 (clone D851308A; kindly provided from Eidia, Japan) at a dilution of 1:3000. Sections were washed three times with phosphate‐buffered saline, pH 7.4 (PBS) for 3 min at 37°C between steps. Antibody binding was visualized by horseradish peroxidase‐labeled goat anti‐mouse or anti‐rabbit IgG (1:100; Dako, Glostrup, Denmark) for 30 min at room temperature. The sections were incubated at room temperature with 3,3′‐diaminobenzidine (DAB) (Dako) after washing three times in PBS to visualize the localization of epitopes. These sections were photographed using a Nikon digital charged‐coupled device camera (DS‐Ri1) mounted on a Nikon E1000M microscope (Nikon, Tokyo, Japan).

Multiple 7‐μm‐thick Frozen transverse section of muscle biopsy specimens from each case were incubated overnight at 4C with primary mouse monoclonal antibodies against MUC1‐C (clone NCL‐MUC1; Novo Castra, Newcastle upon Tyne, UK; 1:100) and KL‐6 (1:5000). Sections were washed three times with PBS for 3 min at 37°C between steps. After incubation, sections were directly visualized with Alexa Fluor 488. For double staining, sections were then incubated with a primary rabbit polyclonal antibody against phosphorylated transactivation response DNA‐binding protein of 43 kDa (TDP‐43) (Cat. No. CAC‐TIP‐PTD‐P01; Cosmo Bio, Tokyo, Japan; 1:3000). After incubation, sections were directly visualized with Alexa Fluor 568. These sections were photographed using a BIOREVO BZ‐9000 Fluorescence Microscope (Keyence, Osaka, Japan).

### Western blotting

Western blotting of muscle biopsy specimens was performed as described.^29^ Extracted tissue proteins were separated with 6.5% sodium dodecyl sulfate‐polyacrylamide gel electrophoresis and electrotransferred onto a nitrocellulose membrane. After blocking with 3% milk in Tris‐buffered saline, pH 7.5 (TBS), consisting of tris(hydroxymethyl)aminomethane and 500 mM NaCl, membranes were probed overnight at 4°C in fresh buffer with the primary mouse antibodies for MUC1‐C (1:1000), KL‐6 (1:1000) and beta‐dystroglycan (Cat. No. H‐242; Santa Cruz Biotechnology, Santa Cruz, CA, USA). Following incubation with a biotinylated secondary antibody, the blots were developed by the chemiluminescence method using the Western Lightning kit (Perkin Elmer, Boston, MA, USA) according to the manufacturer's instructions. The expression of MUC1‐C and KL‐6 was quantified by densitometric analysis with ImageJ 1.46r.

### Quantitative analysis

Statistical analyses were performed using GraphPad Prism 6.0 (GraphPad Software, La Jolla, CA, USA). Values in laboratory data were compared with analysis of variance (ANOVA) followed by post hoc Kruskall—Wallis *H*‐test. In addition, the correlation between serum KL‐6 and patients' characteristics was analyzed using Pearson's correlation coefficient test. A *P*‐value < 0.05 was considered statistically significant.

## RESULTS

### Serum KL‐6 levels were lowest in GNEM patients

**Fig 1:**
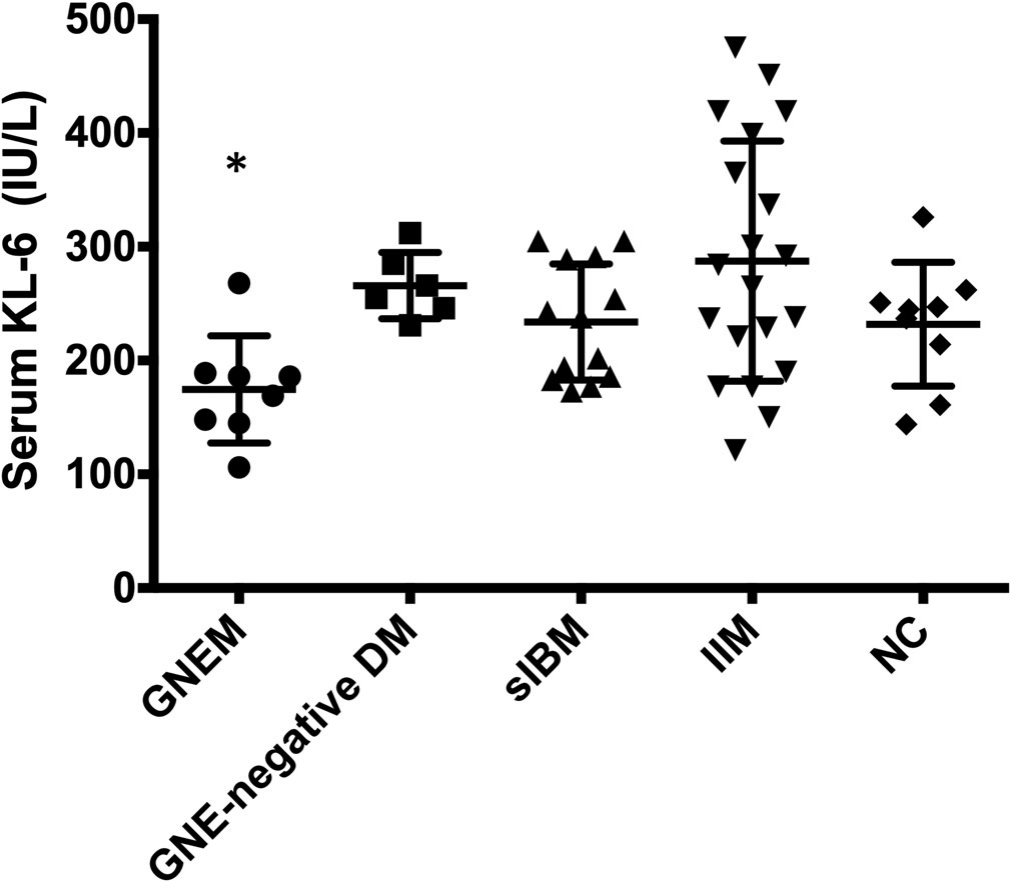
Serum KL‐6 levels in patients of the GNEM, GNE‐negative DM, sIBM, IIM, and NC groups. The levels are the lowest in the GNEM group among all the examined groups. No significant difference in serum KL‐6 levels was found among the groups other than the GNEM group. *P < 0.03.

The characteristics of the study subjects are summarized in Table 1. The patients' profiles and laboratory data, including KL‐6, creatine kinase, and lactate dehydrogenase, are shown in Table 1. Serum KL‐6 levels of the GNEM patients were lower than those of the GNE‐negative DM patients (*P* < 0.03), and the lowest in this study (*P* < 0.03) (Fig. 1). Serum KL‐6 levels of the GNEM patients were correlated with their ages (*P* < 0.01) and were also lower than those of the historical healthy controls that were used to establish the Japanese standard value of serum KL‐6 (mean ± 2 SD; 253 ± 148 IU/L) (*P* < 0.05).^22^ Among the GNEM patients, the serum KL‐6 of patient 7, who was harbouring the homozygous p.D207L mutation, which was rarely observed in GNEM patients, was not lower than those of the GNE‐negative DM patients.

However, there was no significant difference in serum KL‐6 levels among the sIBM, IIM, and NC groups. Serum KL‐6 levels of the IIM patients in this study were the same as those of IIM patients without interstitial pneumonia, as reported previously.^24^ We found no significant correlation between serum KL‐6 and creatine kinase or lactate dehydrogenase.

### MUC1‐C and KL‐6 immunoreactivity was localized in RVs

**Fig 2:**
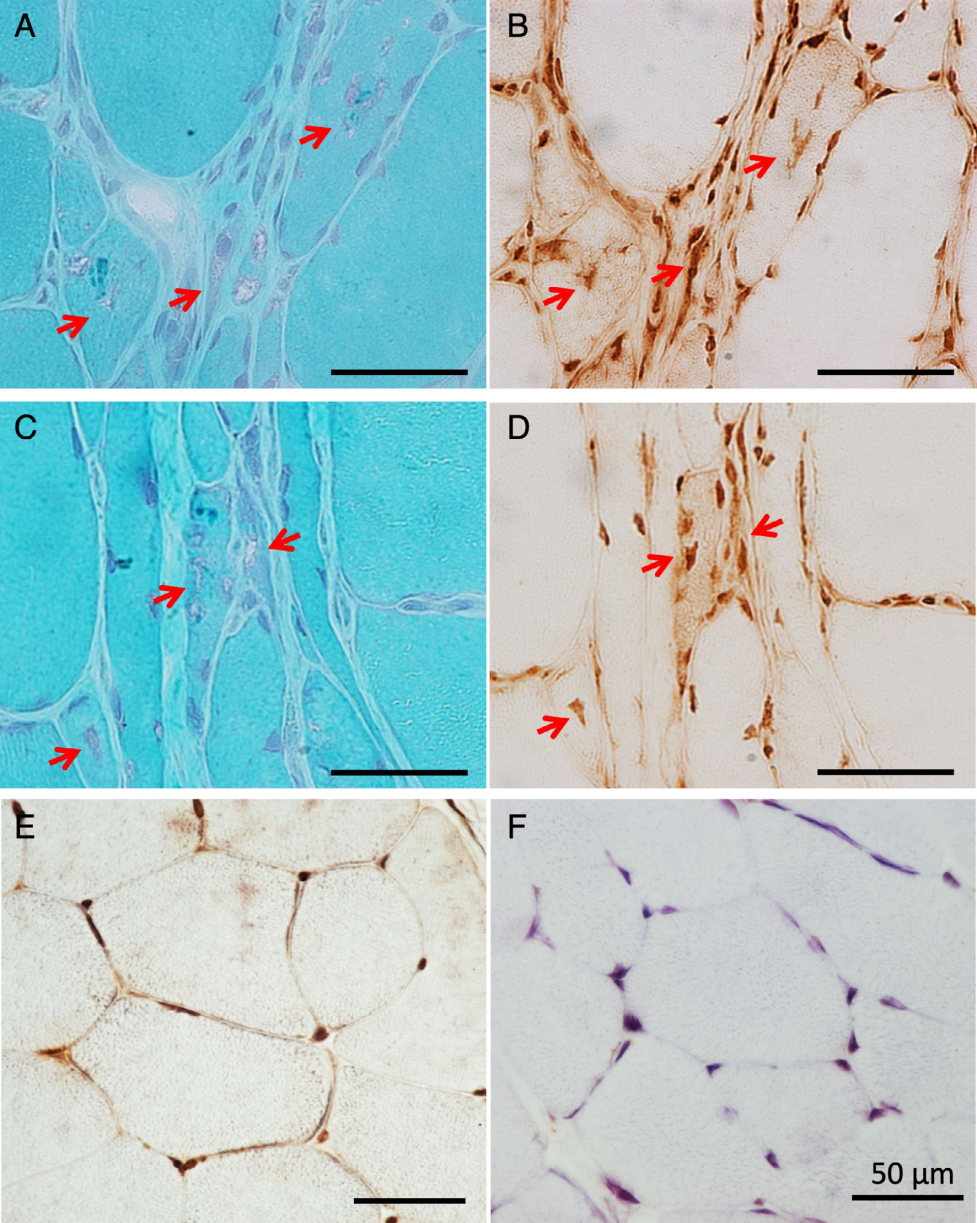
Histological findings in sections of the muscle biopsy specimens. Serial sections show that RVs of GNEM (A) and sIBM (C) cases are positively stained for KL‐6 (B, D). In contrast, IIM (E) and NC (D) cases are negatively stained for KL‐6 in the cytoplasm. Scale bars: 50 μm (A‐F).

**Fig 3:**
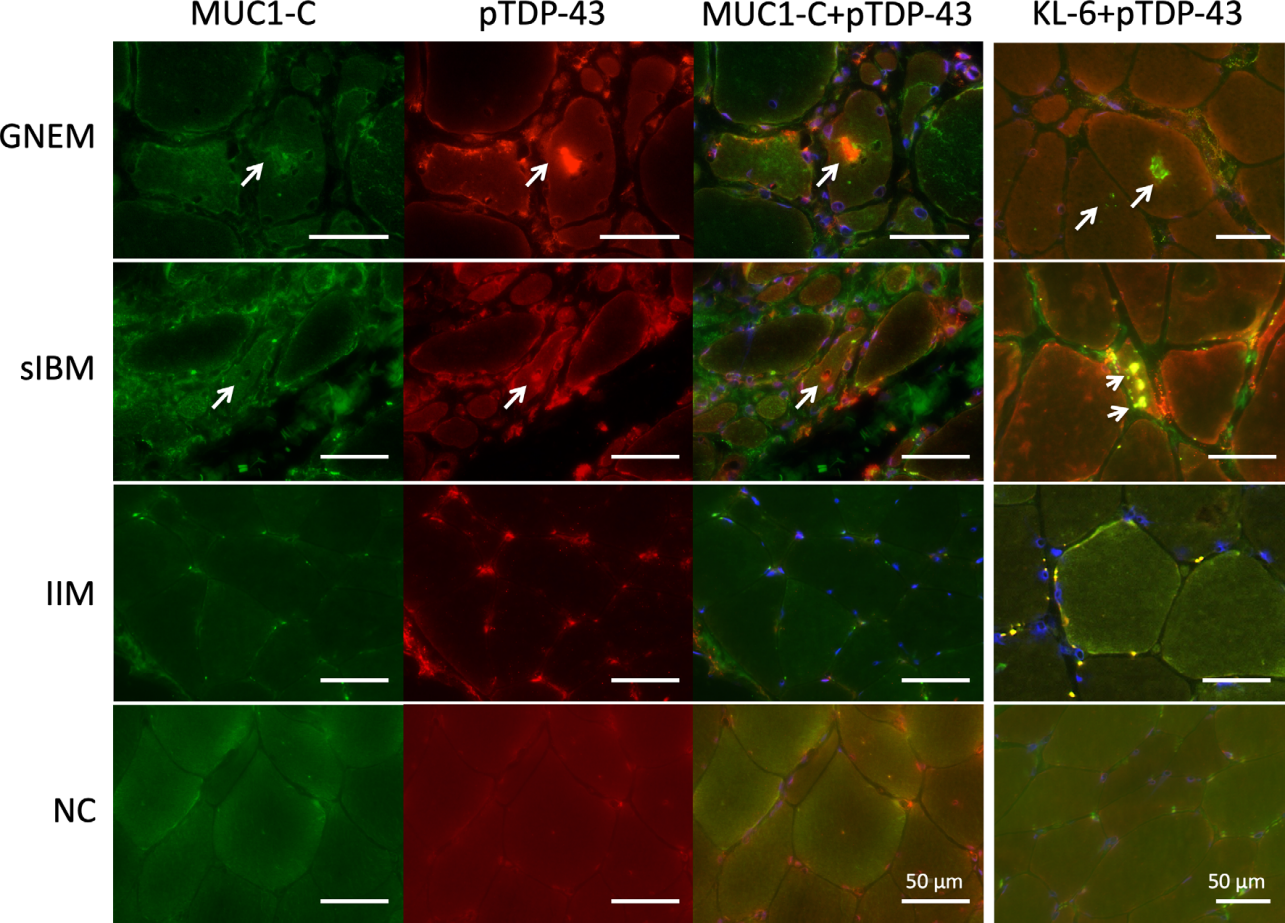
Microphotographs of the muscle biopsy specimen sections stained by the double‐labeling immunofluorescence method. RVs (red), which are positive for phosphorylated TDP‐43 (pTDP‐43) in GNEM and sIBM, are positively stained for MUC1‐C and KL‐6 (green, arrows). In IIM and NC cases, cytoplasmic accumulations are undetectable. Scale bars: 50 μm.

In muscles of the GNEM and sIBM cases, RVs visualized by modified Gomori trichrome staining showed immunoreactivity against KL‐6 (Fig. 2A‐D). In contrast, the IIM and morphologically normal cases showed no cytoplasmic KL‐6 accumulations (Fig. 2E, F). Fluorescence immunohistochemistry also revealed that RVs identified by phosphorylated TDP‐43 were immunorectivity for MUC1‐C and KL‐6 (arrows) in muscles of the GNEM and sIBM cases (Fig. 3 upper). However, the IIM and morphologically normal cases showed no cytoplasmic accumulations that stained for MUC1‐C or KL‐6 (Fig. 3 lower).

### Muscular MUC1‐C and KL‐6 contents decreased in GNEM patients

**Fig 4:**
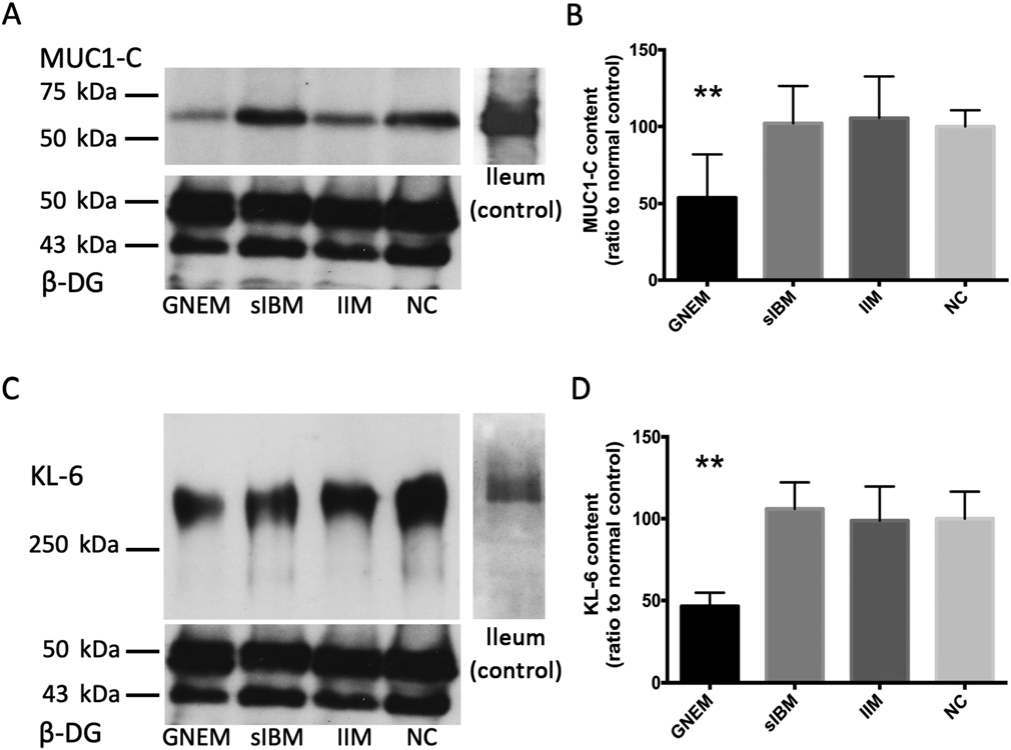
Results of Western blotting (A, C) and densitometry (B, D) for MUC‐C (A, B) and KL‐6 (C, D) in muscle biopsy specimens. (A) MUC1‐C‐immunoreactive bands are observed in the GNEM, sIBM, IIM, and NC groups at the same mobility as the positive control ileum. (B) The normalized MUC1‐C‐immunoreactive signal values are significantly decreased in the GNEM group at approximately 50% of those in the sIBM and NC groups. (C) KL‐6‐immunoreactive bands are observed at a mobility above 250 kDa in the muscles and the positive control ileum. (D) The normalized KL‐6‐immunoreactive signal values are significantly decreased in the GNEM group at approximately 50% of those in other groups. **P < 0.01.

The 50‐kDa MUC1‐C bands were observed in muscle biopsy and ileum specimens. The intensity of MUC1‐C of GNEM were weaker than those of other groups (Fig. 4A). Kruskal–Wallis analysis showed that the MUC1‐C of GNEM were approximately 50% of the MUC1‐C amount of other disease groups (Fig. 4B, *P* < 0.01). KL‐6 bands were also observed above 250 kDa in muscle biopsy and ileum specimens, as previously reported.^18^ The KL‐6‐immunopositive bands (above 250 kDa) of GNEM were also weaker than bands of sIBM, morphologically normal cases, and ileum. In contrast, GNE myopathy cases showed a decrease in KL‐6 (Fig. 4C). KL‐6 levels in GNE myopathy cases were less than 50% of those in sIBM and morphologically normal controls (Fig. 4D, *P* < 0.01).

## DISCUSSION

Hyposialylation due to *GNE* mutations appears to be a major cause of GNEM,^3^, ^4^, ^8^ but the exact pathomechanism is not fully understood.^2^ Previous reports showed that the Thomsen–Friedenreich antigen and transferrin were candidates for evaluating hyposialylation in GNEM.^2^, ^9^, ^10^, ^11^ Here we report that serum and muscle KL‐6 and MUC1 complex levels were decreased in GNEM patients. Serum KL‐6 of GNEM patients was correlated with their ages at biopsy. Western blotting revealed that KL‐6 and MUC1‐C proteins were decreased in GNEM patients, and that MUC1 core proteins were increased in sIBM patients. KL‐6 and MUC1‐C were present in RVs of GNEM and sIBM patients.

MUC1 complexes consist of MUC1 precursor proteins, which are autocleaved in the endoplasmic reticulum to produce MUC1‐C and MUC1‐N. The *VNTR*‐encoded domain of MUC1‐N is glycosylated in the Golgi apparatus.^13^, ^14^ MUC1 complexes are involved in various functions, including inhibition of extracellular interactions and signal transduction.^5^, ^12^ KL‐6 is a sialo‐carbohydrate antigen derived from human MUC1‐N and is detected with a mouse monoclonal antibody recognizing the sialylated sugar chain of its *VNTR*‐encoded domain.^20^ The primary source of serum KL‐6 is type II pneumocytes, especially regenerating type II pneumocytes of humans and apes,^17^, ^20^ and NeuAc accounts for approximately 30% of the KL‐6 sugar content.^18^ Our data showed that MUC1 and KL‐6 are expressed not only in epithelial cells but also in skeletal muscle cells. The *VNTR*‐encoded domains of KL‐6 include high levels of NeuAc.^19^ *GNE* mutations affect not only skeletal muscles but also other organs, including epitheial cells, suggesting that hyposialylation may cause the decrease in KL‐6.^5^, ^7^ As expected, our data showed that serum KL‐6 levels were the lowest in the GNEM group. In addition, serum KL‐6 levels of GNEM patients were lower than those of historical healthy controls, which were used to establish the standard value and to prepare the higher cut‐off line of serum KL‐6.^22^ However, the lower cut‐off line of serum KL‐6 levels has not been prepared for diagnostic use because the lower cut‐off line of serum KL‐6 is considered unnecessary for the diagnosis of interstitial pneumonia.^22^ Further examination is necessary in a large group of GNEM patients and healthy control individuals to establish the specificity and sensitivity of KL‐6 in the GNEM group and the lower cut‐off line of serum KL‐6.

A previous study also reported that serum KL‐6 was associated with aging in normal subjects and patients with interstitial pneumonia.^30^ Our results showed that serum KL‐6 levels of GNEM patients were correlated with their ages, and that serum KL‐6 levels of patient 7 was not lower than those of other groups. Among GNEM patients, patient 7 harboured the homozygous p.D207L mutation. Homozygous p.D207L mutation decreases the enzymatic activity of GNE more mildly than other *GNE* mutations and has no effect on the MNK enzymatic activity.^7^ In contrast, the p.V603L mutation decreases the enzymatic activity of MNK, the defect of which causes more severe hyposialylation than that of GNE. The difference in serum KL‐6 levels between patient 7 and other GNEM patients might present in the severities of their hyposialylation. Further examinations were needed to clarify the association between serum KL‐6 levels and the hyposialylation.

MUC1‐C directly regulates the expression of metabolic genes, and modulates ErbB family proteins, including the epidermal growth factor receptor (EGFR) and human EGFR 2 (HER2).^31^, ^32^, ^33^ In the presence of MUC1‐C, endocytosed EGFR and MUC1‐C undergo nuclear trafficking and accumulation, which are necessary for active transcription following EGF stimulation. In the absence of MUC1‐C, EGFR is exported from the nucleus and degraded in the lysosome. These alterations may be necessary to drive tumor progression in EGFR‐dependent tumors.^31^ KL‐6 is also a chemotactic factor for fibroblasts and promotes proliferation and survival of fibroblasts by suppressing apoptosis, leading to fibrosis in interstitial pneumonia.^20^, ^22^ The proliferative and anti‐apoptotic effects of KL‐6 are greater than those of any other growth factors.^22^, ^32^ Our results showed that MUC1 and KL‐6 existed in RVs of GNE myopathy and sIBM. Our observations suggested that EGFR with KL‐6 might be involved in endocytotic processes of EGFR, which did not form complexes with MUC1‐C caused by hyposialylation.

Mutations in the VNTR sequence of *MUC1* produce a large *VNTR*‐encoded domain and are associated with the absence of MUC1‐C. The lack of MUC1‐C inhibits transcription that is normally upregulated by EGFR and HER2 activation. Despite degradation of ErbB family proteins, mutated MUC1‐N with a large *VNTR*‐encoded region causes renal fibrosis.^33^, ^34^ Our data showed that MUC1‐C and KL‐6 were decreased in muscle biopsy specimens from patients with GNEM. In addition, in GNEM, RVs were immunoreactive for MUC1‐C and KL‐6. These findings suggested that the lack of KL‐6 might be associated with abnormal autophagocytosis due to decreased MUC1‐C and reduced transcriptional upregulation.

In conclusion, serum and muscle levels of KL‐6 and MUC1 were decreased in GNEM patients, and KL‐6 and MUC1 were not localized in the sarcolemma but rather in RVs of specimens from patients with GNEM. KL‐6 may be associated with the pathogenesis of GNEM. Because KL‐6 is already used to evaluate lung diseases, KL‐6 may be a novel candidate for evaluation of hyposialylation in GNEM.

## DISCLOSURE

The authors declare that they have no conflict of interest regarding the publication of this paper.

## Supporting information


**Table S1.** Clinical characteristics in this study
