# Supplementary material for: Krebs von den Lungen 6 decreased in the serum and muscle of GNE myopathy patients
Source: Neuropathology. 2020 Nov 22;41(1):29–36. doi: 10.1111/neup.12703 (PMC7983952; doi:10.1111/neup.12703)
Supplement: Supplementary file 1 — Table S1. Clinical characteristics in this study [file NEUP-41-29-s001.docx]

| *Table 1. Clinical characteristics in this study* | | | | | | | | |
| --- | --- | --- | --- | --- | --- | --- | --- | --- |
| No. | Diagnosis | Age at biopsy  (years) | Sex | Disease duration  (years) | Biopsy  site | Serum  KL-6 (IU/L) | Serum  CK (IU/L) | Serum  LDH (IU/L) |
| 1 | GNEM (p.V603L/p.V603L) | 31 | M | 9 | Lt. Quad | 145 | 518 | 465 |
| 2 | GNEM (p.V603L/p.V603L) | 37 | F | 11 | Lt. TA | 189 | 531 | 221 |
| 3 | GNEM (p.V603L/p.V603L) | 18 | F | 3 | Lt. TA | 106 | 68 | 173 |
| 4 | GNEM (p.V603L/p.V603L) | 33 | M | 10 | Lt. TA | 173 | 444 | 192 |
| 5 | GNEM (p.V603L/p.V603L) | 38 | M | 5 | Lt. BB | 186 | 664 | 221 |
| 6 | GNEM (p.V603L/p.V603L) | 29 | M | 7 | Lt. TA | 148 | 210 | 419 |
| 7 | GNEM (p.D207L/p.D207L) | 63 | M | 3 | Lt. BB | 268 | 736 | 236 |
| 8 | GNEM (p.G167R/p.D207L) | 30 | M | 8 | Lt. BB | 169 | 1910 | 285 |
| 9 | GNE (-) DM | 44 | F | 10 | Lt. BB | 266 | 736 | 394 |
| 10 | GNE (-) DM | 33 | F | 16 | Rt. BB | 231 | 674 | 421 |
| 11 | GNE (-) DM | 57 | M | 45 | Lt. Delt | 312 | 165 | 168 |
| 12 | GNE (-) DM | 27 | F | 14 | Lt. BB | 246 | 314 | 239 |
| 13 | GNE (-) DM | 37 | F | 12 | Lt. BB | 255 | 2263 | 562 |
| 14 | GNE (-) DM | 49 | M | 22 | Lt. BB | 285 | 29 | 139 |
| 15 | sIBM | 80 | F | 4 | Lt. BB | 305 | 1163 | 585 |
| 16 | sIBM | 84 | M | 6 | Rt. Quad | 183 | 1046 | 447 |
| 17 | sIBM | 49 | F | 4 | Rt. Quad | 173 | 192 | 245 |
| 18 | sIBM | 81 | F | 2 | Rt. Quad | 202 | 218 | 190 |
| 19 | sIBM | 57 | M | 1 | Lt. BB | 238 | 1379 | 228 |
| 20 | sIBM | 78 | F | 4.5 | Lt. RF | 305 | 1163 | 585 |
| 21 | sIBM | 76 | M | 3 | Rt. Quad | 254 | 299 | 277 |
| 22 | sIBM | 51 | M | 3 | Rt. BB | 194 | 768 | 308 |
| 23 | sIBM | 44 | M | 3.5 | Lt. BB | 291 | 456 | 911 |
| 24 | sIBM | 69 | M | 3 | Lt. BB | 177 | 586 | 312 |
| 25 | sIBM | 61 | M | 2 | Lt. RF | 186 | 1733 | 644 |
| 26 | sIBM | 71 | M | 2 | Lt. RF | 243 | 840 | 438 |
| 27 | sIBM | 57 | M | 2 | Lt. VL | 289 | 586 | 312 |
| 28 | IIM | 57 | M | 0.5 | Lt. Quad | 150 | 5136 | 1756 |
| 29 | IIM | 72 | F | 2 | Lt. Quad | 284 | 5139 | 1021 |
| 30 | IIM | 60 | F | 1 | Lt. BB | 177 | 1031 | 615 |
| 31 | IIM | 24 | F | 0.5 | Rt. Quad | 121 | 13850 | 465 |
| 32 | IIM | 59 | M | 1 | Lt. BB | 475 | 1876 | 460 |
| 33 | IIM | 70 | M | 1 | Lt. Quad | 451 | 4051 | 821 |
| 34 | IIM | 54 | M | 1 | Lt. Quad | 238 | 1513 | 627 |
| 35 | IIM | 59 | F | 1 | Lt. BB | 221 | 2628 | 497 |
| 36 | IIM | 57 | M | 0.1 | Lt. VL | 419 | 319 | 215 |
| 37 | IIM | 51 | F | 1 | Lt. Quad | 229 | 9705 | 1500 |
| 38 | IIM | 71 | F | 0.5 | Lt. VL | 190 | 2796 | 1106 |
| 39 | IIM | 62 | F | 0.5 | Lt. VL | 399 | 268 | 247 |
| 40 | IIM | 82 | M | 0.5 | Lt. BB | 337 | 1056 | 396 |
| 41 | IIM | 67 | F | 1 | Lt. BB | 365 | 521 | 3116 |
| 42 | IIM | 75 | F | 0.5 | Lt. BB | 237 | 796 | 490 |
| 43 | IIM | 60 | F | 1 | Lt. BB | 177 | 1031 | 615 |
| 44 | IIM | 71 | M | 1 | Lt. VL | 265 | 520 | 319 |
| 45 | IIM | 40 | F | 7 | Lt. TB | 301 | 2059 | 559 |
| 46 | IIM | 57 | F | 3 | Lt. TB | 419 | 319 | 215 |
| 47 | IIM | 62 | M | 2 | Lt. VL | 292 | 685 | 2896 |
| 48 | NC | 35 | F |  | Lt. VL | 237 | 1305 | 334 |
| 49 | NC | 38 | M |  | Lt. VL | 247 | 7432 | 553 |
| 50 | NC | 23 | M |  | Lt. BB | 144 | 211 | 183 |
| 51 | NC | 32 | F |  | Lt. BB | 214 | 187 | 124 |
| 52 | NC | 17 | M |  | Rt. FB | 161 | 147 | 150 |
| 53 | NC | 31 | F |  | Lt. BB | 251 | 23322 | 2200 |
| 54 | NC | 30 | F |  | Lt. VL | 326 | 276 | 607 |
| 55 | NC | 37 | M |  | Lt. FB | 262 | 93 | 360 |
| 56 | NC | 28 | M |  | Lt. BB | 245 | 104 | 161 |

Abbreviations: KL-6, Krebs von den Lungen-6; CK, creatine kinase; LDH, lactate dehydrogenase; GNEM, GNE myopathy; sIBM, sporadic inclusion body myositis; IIM, idiopathic inflammatory myopathies; NC, morphologically normal control; Lt., left; Rt., right; Delt, Deltoid muscle; Quad, quadriceps femoris muscle; TA, tibialis anterior muscle; BB, biceps brachii muscle; RF, rectus femoris muscle; VL, vastus lateralis muscle; FB, fibularis brevis muscle; TB, triceps brachii muscle.
